# Freedom to Ride, Responsibility to Insure: Florida’s Motorcycle Trauma Coverage Gap When Private Risk Produces Publicly Financed Rescue

**DOI:** 10.7759/cureus.114941

**Published:** 2026-08-21

**Authors:** Andrew M Klapper, Anthony N Dardano, Michael Risin, Mariella Moreno, Zachary Ehrlich

**Affiliations:** 1 Plastic and Reconstructive Surgery, Delray Medical Center, Delray Beach, USA; 2 Plastic and Reconstructive Surgery, Larkin Community Hospital Palm Springs Campus, Miami, USA; 3 Medicine, University of Central Florida College of Medicine, Orlando, USA

**Keywords:** cost shifting, health policy, helmet legislation, insurance mandates, motorcycle trauma, personal injury protection, trauma systems, uncompensated care

## Abstract

Florida’s motorcycle insurance framework can separate individual risk-taking from the financing of catastrophic trauma care. When meaningful first-party medical coverage is absent or inadequate, unreimbursed costs may be shifted to hospitals, physicians, public programs, insured families, and taxpayers. This editorial argues that the resulting arrangement can function as an implicit subsidy of a privately chosen motor-vehicle risk and reviews historical Florida and contemporary out-of-state evidence describing that potential burden. Because the current statewide magnitude, payer distribution, and uncompensated component of Florida motorcycle trauma are not publicly quantified, the editorial proposes a two-step legislative response: first, systematic measurement of Florida’s motorcycle trauma coverage deficit through trauma-registry reporting; and second, selection of a financing remedy: mandatory first-party coverage, risk-adjusted limits for helmetless riders, verified comprehensive health coverage, or a dedicated trauma fund sized to the measured gap. The argument is not against riding or helmet choice. It is against separating freedom from its foreseeable financial consequences.

## Editorial

Consider a composite case, drawn from years in Florida trauma bays rather than from any single patient. The helicopter calls ahead. A young rider arrives without a helmet, hypotensive, and with an open femur fracture. A fully staffed trauma team converges before he can say his own name. Everyone in that room is bound by law, by training, and by conscience to save him, whatever it costs.

Nothing in Florida’s motorcycle-registration framework reliably required him to carry meaningful first-party coverage for the care he was about to receive [1]. In this editorial, meaningful first-party coverage means coverage available without awaiting fault determination and sufficient to finance a material share of the rider’s acute and post-acute care.

Ask almost anyone in Florida who should pay after a catastrophic motorcycle crash (defined here as one requiring trauma-center admission, major operative treatment, intensive care, or prolonged rehabilitation), and the answer seems obvious: the rider and the rider’s insurer. That is what personal responsibility is supposed to mean. It is not how Florida works. Here, a rider can sustain devastating injuries, generate hundreds of thousands of dollars in hospital and physician costs, and discover that the insurance policy attached to the motorcycle may have no contractual obligation to pay for his own medical care. The rider may be unable to pay. The motorcycle policy may contain no first-party medical obligation [1]. Participating hospitals cannot refuse mandated emergency screening and stabilization. The balance may be pushed outward onto health insurers, Medicaid, hospital charity care, unpaid physicians, commercial premiums, and ultimately Florida taxpayers and insured families [2-5].

This is not an insurance technicality. It is a state-enabled transfer of private risk onto the public.

The coverage gap

Florida excludes motorcycles from the Personal Injury Protection system that governs automobiles [1]. As a result, a motorcycle may be “insured” in the ordinary sense - typically for bodily-injury liability. The policy may protect those injured by the rider while providing little or no coverage for the rider’s own injuries. In practical terms: the machine is insured. The rider may not be [1].

That distinction is poorly understood, and it is central to the policy failure. The existence of a motorcycle policy suggests financial responsibility, yet the policy may do little to finance the rider’s own catastrophic trauma care. A rider can hold first-party protection - optional medical payments coverage, health insurance, uninsured motorist benefits when another driver is at fault - but Florida does not require it, and nothing guarantees it is there when the helicopter lands [1].

Florida’s celebrated helmet freedom rests on the same thin foundation: adults aged 21 years and older may ride without helmets if covered by at least $10,000 in qualifying medical benefits [1]. In a catastrophic trauma admission, $10,000 can be consumed before the first operation ends. Indeed, in early-2000s dollars, roughly three-quarters of hospital-admitted riders with head, brain, or skull injuries after Florida’s 2000 repeal incurred reported hospital charges - not necessarily economic costs - exceeding $12,000 [2], above a statutory threshold that has not changed in the two decades since. Even against trauma charges from more than 20 years ago, the safeguard was already obsolete. It is a rounding error dressed up as responsibility. Reported hospital charges are not equivalent to economic cost or amounts ultimately paid, but they illustrate how quickly a nominal $10,000 benefit can become inadequate in a serious trauma admission.

The result is the appearance of financial accountability without its substance. The state protects the rider’s freedom to assume the risk but does not systematically verify through registration that coverage proportionate to the foreseeable cost of that risk exists. The choice stays personal. The bill goes public [1,2].

The crash is only the beginning

A catastrophic motorcycle crash does not produce an ordinary medical bill. It activates an entire system within minutes: emergency physicians, trauma and neurosurgeons, orthopedic and reconstructive surgeons, anesthesiologists, intensive-care teams, the blood bank, operating rooms. And the meter does not stop when the bleeding does. Severe motorcycle injuries mean prolonged intensive care, repeated operations, complex limb and soft-tissue reconstruction, rehabilitation, readmissions, and often lifelong disability support. These are among the most resource-intensive trauma episodes any center treats - and the insurance product most directly connected to the injury may contribute nothing at all [2-4].

Motorcycle insurers are not required to assume that exposure; the trauma system cannot decline it. The crash is the spark; the financing system decides who absorbs the fire [1,5].

Freedom without financial responsibility is not freedom - it’s a subsidy

Let me be clear about what this argument is not. It is not an attack on riding, and it is not a case for mandatory helmets. Adults should be free to weigh risk against joy, independence, and the open road, and many Floridians rightly cherish that freedom. Those decisions belong to the rider.

But the financial consequences should belong to the rider too, and in Florida they largely don’t. Freedom and financial responsibility are two halves of the same principle; the state has severed them and kept only the popular half. This functions as an implicit subsidy in the economic sense: part of the cost of the activity is transferred from the person choosing the risk to hospitals, clinicians, public programs, insured families, and taxpayers who neither assumed nor priced that risk. No direct government payment is required for a subsidy to exist - only an uncompensated transfer. A liberty whose predictable costs are quietly billed to strangers is not liberty. It is a subsidy with a leather jacket on.

An obvious objection deserves a direct answer: many activities impose costs on others - skiing, horseback riding, smoking - so why single out motorcycles? Because motorcycling is not merely a risky pastime the state has declined to regulate. It is a state-created exception to a financial-responsibility regime the state already operates. Florida long ago decided that operating a motor vehicle on public roads carries a duty to secure coverage, then exempted from the medical core of that regime a vehicle class associated with unusually severe injury when crashes occur. The argument here does not ask government to police private risk generally. It asks Florida to stop exempting one category of motor-vehicle operation from a principle it already applies to all the others [1-4].

An expensive risk does not disappear

Insurers understand risk better than almost anyone. They price frequency, severity, vehicle type, and expected claims with actuarial precision. So when catastrophic motorcycle medical coverage is expensive, that is not a market failure - it is the market telling the truth. The underlying risk is expensive. Exempting riders from buying the coverage does not make the risk cheaper. It only changes who pays.

Because participating hospitals with emergency departments cannot opt out of the Emergency Medical Treatment and Labor Act's (EMTALA’s) screening and stabilization requirements [5], the emergency department cannot decline the patient because the injury is costly. The trauma surgeon cannot stop operating because the policy is exhausted. The reconstructive surgeon cannot abandon an open fracture because the motorcycle policy excludes medical coverage. The risk the insurance transaction rejects is transferred to the hospital by default. Whatever that is, it is not a free market. A free market requires the person choosing the risk to buy the protection that finances it.

The hidden subsidy, measured elsewhere

This burden was already measurable more than two decades ago. In its 2005 evaluation of Florida’s 2000 helmet-law repeal, the National Highway Traffic Safety Administration analyzed crash and hospital data from the early 2000s. It found that motorcyclist fatalities rose 81% over the following three years, compared with 48% nationally; hospital admissions for head injuries rose 82%; and the average reported charge for treating a head injury increased by nearly $10,000, to more than $45,000 [2]. These are historical, unadjusted figures - not current trauma costs - yet even then the average charge exceeded Florida’s $10,000 statutory medical benefit threshold by more than fourfold. For approximately one in five hospital-admitted motorcyclists in that evaluation, a combined $10.5 million in costs over 30 months was billed to charitable and public sources, including Medicaid [2]. National trauma-registry data show that unhelmeted riders were more likely than helmeted riders to be government-insured or uninsured and to sustain more severe injuries [3]. These are associations and do not establish that helmet status causes payer status; socioeconomic, demographic, behavioral, and crash-related factors may contribute to the observed relationships. And when Michigan repealed its universal helmet law, average inpatient costs per motorcycle crash patient rose by $5,785 - a 26% increase [4]. Yet none of these sources measures the quantity this argument most needs: the current magnitude, payer distribution, and uncompensated component of motorcycle trauma in Florida. No statewide accounting of that burden appears to be publicly available. This is a principal limitation of the existing evidence and prevents quantification of the present deficit. It should not be interpreted as evidence that the burden does not exist; it demonstrates why Florida should measure it.

National data show that unhelmeted riders are more likely to be uninsured or covered by government programs, while Florida’s historical evaluation documented substantial motorcycle trauma charges billed to charitable and public sources [2,3]. When available coverage is absent or inadequate, the remaining costs can fragment across hospital bad debt, uncompensated professional services, and public programs.

No Floridian will ever see a line item labeled “motorcycle trauma subsidy.” The subsidy exists anyway. It is simply hidden - and invisible costs are the easiest costs for lawmakers to ignore.

**Figure 1:**
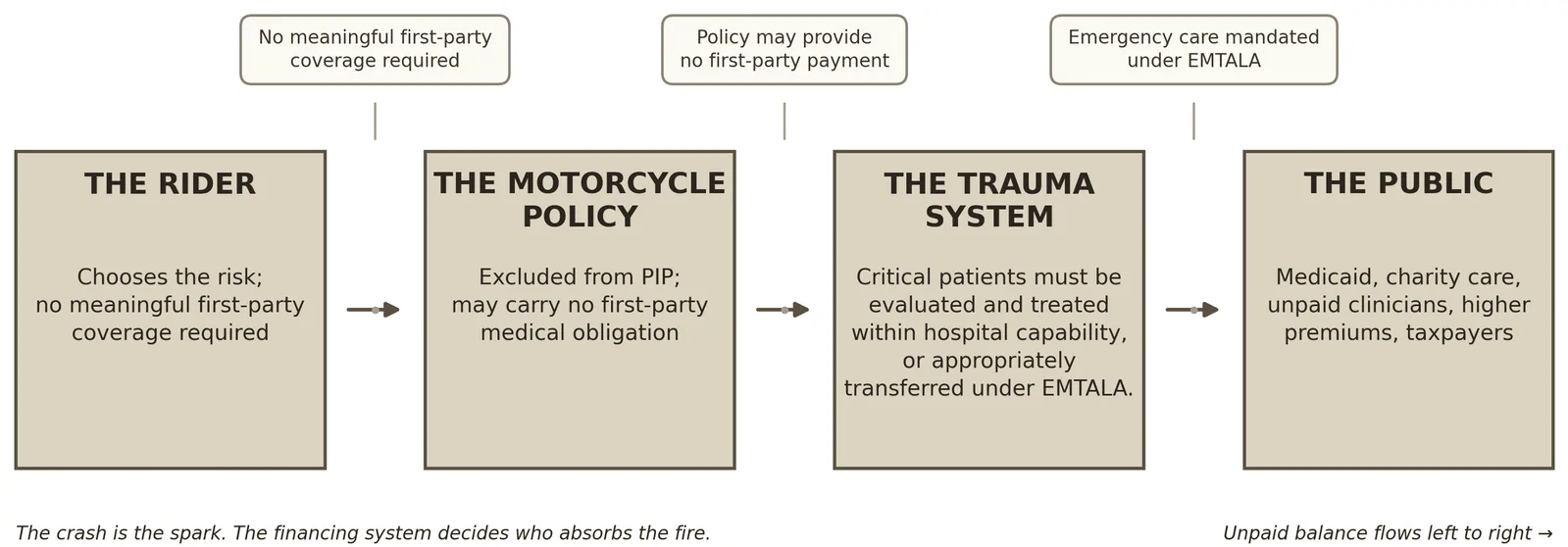
The cost cascade of catastrophic motorcycle trauma in Florida The cost cascade of catastrophic motorcycle trauma in Florida. The rider chooses the risk without a meaningful first-party coverage requirement; the motorcycle policy, excluded from Personal Injury Protection, may carry no first-party medical obligation; the Emergency Medical Treatment and Labor Act (EMTALA) requires participating hospitals with emergency departments to provide an appropriate medical screening examination and, when an emergency medical condition is identified, stabilizing treatment or an appropriate transfer [5]; and the unpaid balance fragments across Medicaid, hospital charity care, uncompensated clinicians, commercial premiums, and taxpayers. PIP: Personal Injury Protection Source: Authors’ original conceptual model informed by references [1-5].

Against this backdrop, predictable, severe, and resource-intensive injuries can enter the healthcare system without a reliable first-party payment source. The result is selective cost shifting: the rider enjoys the freedom, the insurer avoids the exposure, the trauma team absorbs the work, and the public absorbs the deficit [2-4] (Figure 1).

This is not an argument to deny care

No injured motorcyclist should ever be turned away. That principle is not negotiable, and no trauma professional would have it otherwise. We do not ask whether the patient made a wise decision before controlling hemorrhage, securing an airway, decompressing a brain, or rebuilding a shattered limb. We treat the patient.

Public safety nets are appropriately designed to preserve access when patients cannot pay. The policy question is not whether those protections should exist, but whether Florida should rely on general safety-net programs while exempting this category of motor-vehicle risk from meaningful first-party financing.

That is precisely why financing must be settled before the crash. Under the EMTALA (42 U.S.C. § 1395dd), participating hospitals with emergency departments must provide an appropriate medical screening examination and, when an emergency medical condition is identified, stabilizing treatment or an appropriate transfer [5]. Professional ethics demand no less. Society has correctly decided that rescue is mandatory. If rescue is mandatory, financing cannot remain optional. The remedy is not to punish an injured rider at the bedside; it is to require adequate financial preparation before the motorcycle ever reaches the road.

First, measure the gap

The state cannot manage a cost it refuses to measure, and the federal snapshot from two decades ago is no substitute for current, Florida-specific accounting. Florida’s trauma registries and hospitals should report, for every serious motorcycle crash: helmet status, motorcycle and health insurance status, available first-party coverage, hospital and professional charges, payments actually received, uncompensated care, rehabilitation needs, and subsequent enrollment in public programs. From those data, Florida could calculate its motorcycle trauma coverage deficit: the difference between (1) documented episode-level hospital and professional resource costs, rather than gross charges, and (2) total payments actually received from all sources. The state’s published methodology should define the episode window, cost-accounting method, treatment of multiple payers and subsequent public coverage, and handling of later third-party recoveries. The state should publish the resulting figure annually together with its methodology and principal limitations.

Then, choose a remedy

With the deficit quantified, Florida can preserve the freedom and demand the responsibility, selecting among financing mechanisms sized to the measured gap (Table 1). The options are not mutually exclusive: a verification requirement enforces the coverage condition Florida’s helmet statute already imposes, while a trauma fund can backstop whatever individual coverage misses [1-5].

**Table 1:** Candidate financing mechanisms for catastrophic motorcycle trauma in Florida Comparison of four policy approaches that could reduce the motorcycle trauma coverage deficit while preserving rider choice. Each option is summarized by its proposed design, principal strengths, and implementation considerations. The mechanisms are presented for comparative policy analysis and are not mutually exclusive. Source: Authors’ original comparative policy synthesis informed by references [1-5].

Mechanism	Design	Strengths	Limitations and design cautions
Mandatory first-party medical coverage	Every registered motorcycle carries meaningful medical payments coverage scaled to realistic trauma costs; proof verified at registration and renewal.	Aligns financing directly with the risk-creating activity; uses the existing insurance market; payment certainty at admission.	Premium cost may burden lower-income riders and invite noncompliance; requires setting and updating an adequate minimum limit.
Risk-adjusted limits for helmetless riders	Riders exercising the helmet exemption carry higher minimum medical benefits, reflecting greater expected injury severity and cost.	Prices the specific choice that raises expected cost; fully preserves the choice itself; actuarially coherent.	Requires actuarially defensible tiering; roadside verification is harder than registration-based verification.
Verified comprehensive health coverage	Riders may instead demonstrate health insurance that genuinely covers motorcycle injuries - not a nominal benefit exhausted within hours - with verification at registration.	Avoids duplicate coverage; effectively enforces the condition the existing helmet statute already states; minimal new mandate.	Plan adequacy varies (deductibles, networks, rehabilitation limits); coverage can lapse between registration cycles.
Motorcycle trauma fund	A dedicated fund - financed by registration fees, insurer assessments, or a helmetless-riding surcharge - reimburses trauma centers and clinicians for otherwise uncompensated motorcycle trauma care.	Spreads cost across the riding population through modest fees; directly compensates the safety net; most responsive to equity concerns.	Fees may be characterized as taxation; requires administration and solvency oversight; weakest individual incentive alignment.

These are policy options, not claims of proven effectiveness in Florida. Any adopted mechanism should coordinate benefits and subrogation with existing coverage to prevent duplicate payment, and its effect should be evaluated by measuring changes in uncompensated hospital and professional care.

Any mandate must also reckon honestly with equity. Actuarially fair coverage will be expensive precisely because the risk is, and a poorly designed requirement could price out lower-income riders or push them into noncompliance. That argues for careful design, not inaction: graduated limits, group purchasing, or the trauma-fund model, which spreads costs across the riding population rather than concentrating them in individual premiums. The distributional choice is therefore not between imposing costs and avoiding them, but between leaving residual costs with patients, safety-net providers, clinicians, and public programs or financing them more directly through riders, insurers, or a rider-funded pool.

One further objection warrants a direct answer: in many multi-vehicle motorcycle crashes the other driver is at fault, so why should the rider pre-finance injuries caused by someone else’s negligence? Because in Florida, planning to recover from the at-fault driver is planning by wishful thinking. Florida law generally does not condition private vehicle registration on bodily injury liability coverage [1]. The car that strikes a motorcyclist may therefore be driven, lawfully, by someone with no bodily injury liability coverage and no assets worth pursuing. Fault determination takes months; collection from an uninsured or judgment-proof driver may take forever; the trauma center incurs costs at hour one. First-party coverage is not a concession of blame - it is financing certainty at the moment of rescue, with subrogation available to pursue the at-fault driver afterward. This is precisely the logic Florida itself adopted when it built a no-fault system for automobiles: medical bills cannot wait for a verdict. The rider is not being asked to accept fault. He is being asked to stop wagering his trauma care on the insurance decisions of strangers.

The mechanism is open to debate. The principle is not: the financial burden must stay as close as possible to the activity that creates the risk.

Your choice, your risk, your obligation to insure it

Motorcycling means freedom to many Floridians, and that freedom deserves respect. But freedom does not mean choosing the risk and assigning the consequences to everyone else. Florida today lets motorcycles operate outside the no-fault medical structure, lets adults decline helmets against a token financial standard, and does not systematically verify through registration that meaningful catastrophic coverage exists before the crash [1]. The predictable deficit lands on hospitals, physicians, public programs, insured families, and taxpayers [2-4]. That is not personal responsibility. It is risk externalization.

The principle should be simple: ride if you choose, and decline the helmet if the law permits, but maintain a credible means of financing the foreseeable consequences of that decision.

When rescue is mandatory, financing cannot remain optional.
